# Outcomes in Dogs with Multiple Sites of Cervical Intervertebral Disc Disease Treated with Single Ventral Slot Decompression

**DOI:** 10.3390/vetsci10060377

**Published:** 2023-05-28

**Authors:** Ya-Pei Chang, Wei-Hsiang Huang, Wan-Zhen Lua, Wenyi Wong, I-Hsuan Liu, Chen-Hsuan Liu

**Affiliations:** 1 Department and Graduate Institute of Veterinary Medicine, School of Veterinary Medicine, National Taiwan University, Taipei 106319, Taiwan; 2 Graduate Institute of Veterinary Clinical Science, School of Veterinary Medicine, National Taiwan University, Taipei 106319, Taiwan; 3 National Taiwan University Veterinary Hospital, National Taiwan University, Taipei 106328, Taiwan; 4 Graduate Institute of Molecular and Comparative Pathobiology, School of Veterinary Medicine, National Taiwan University, Taipei 106319, Taiwan; 5 Department of Animal Science and Technology, National Taiwan University, Taipei 106073, Taiwan; 6 Research Center for Developmental Biology and Regenerative Medicine, National Taiwan University, Taipei 106038, Taiwan

**Keywords:** canine, decompression, degenerative, disc extrusion, disc protrusion, intervertebral disc, multiple discs, surgery, ventral slot

## Abstract

**Simple Summary:**

The typical clinical manifestation of intervertebral disc disease (IVDD) in dogs is an acute disc extrusion leading to spinal cord injury. However, imaging investigations occasionally reveal spinal cord compression associated with multiple sites of IVDD in dogs with an acute presentation. In this scenario, one acute disc extrusion is likely accompanied by previously extruded or protruded discs. Currently, its most appropriate management remains unclear. Here we described the outcomes and investigated prognostic factors in 40 dogs with an acute clinical presentation but diagnosed with multiple sites of spinal cord compression from cervical disc extrusion or protrusion on MRI who underwent surgical decompression for the single acute disc. The overall recovery rate was 97.5%. The median recovery time was seven days. Compared to 23 dogs with single disc extrusion treated surgically, the recovery outcomes were similar between dogs with multiple-site IVDD and single disc extrusion. Moreover, the total number of IVDDs with concomitant spinal compression did not influence the recovery time or outcomes. If an acute disc site could be identified, the described management strategy could be a practical approach for dogs with an acute presentation but diagnosed with multiple sites of spinal cord compression from IVDD.

**Abstract:**

In dogs with acute myelopathy but showing multiple sites of spinal compression from intervertebral disc disease (IVDD) on imaging, one approach is surgical decompression of the single acute disc extrusion while ignoring other previously extruded or protruded discs. However, little is known regarding the outcomes of this approach. This study described the outcomes and investigated prognostic factors in 40 dogs with multiple sites of cervical disc extrusion or protrusion on MRI who underwent ventral slot decompression for the single acute disc. The overall recovery rate was 97.5%. The median recovery time was seven days. The number of affected discs (including disc extrusion and protrusion) and the presence and number of the affected discs causing severe spinal compression did not influence the 30-day outcome. Compared with 23 dogs with single disc extrusion treated surgically, the recovery time and outcomes were similar between the two groups. The total number of affected discs was not associated with recovery time or outcomes. In conclusion, if an acute disc could be identified, ventral slot decompression targeting the single acute disc is a viable management approach for dogs with an acute presentation but diagnosed with multiple sites of spinal cord compression from IVDD.

## 1. Introduction

Intervertebral disc disease (IVDD) is a common condition leading to spinal cord injury in dogs. Several subtypes, such as degenerative intervertebral disc extrusion (IVDE) and intervertebral disc protrusion (IVDP), have been described since the early 1950s [1]. The most common clinical manifestation of IVDD is a single acute IVDE that leads to acute spinal pain and/or myelopathy [2,3]. The incidence rate is higher in chondrodystrophic breeds. In all dogs with IVDE, approximately 16–25% of cases affect the cervical region [4,5]. In a study describing cervical and thoracolumbar myelopathy caused by IVDD in dogs in Japan, the distribution of cervical and thoracolumbar discs was approximately 1:1.25 [6].

Although a single IVDE leading to acute myelopathy is the most common clinical manifestation, in some patients with acute clinical signs, imaging investigations may reveal multiple sites of IVDD leading to spinal cord compression. In this scenario, there is likely a single acute IVDE accompanied by chronic IVDP or sub-clinical IVDE that caused minimal or unnoticed clinical signs in the past [2]. Alternatively, the recurrence of clinical signs consistent with IVDE was also well documented [7,8]. A recent study demonstrated that, following successful medical treatment of cervical IVDE, a recurrence of clinical signs was noted in 36% of dogs, of which 50% of dogs showed recurrence at a different disc site [9]. One management approach for the above situation is to identify the single disc that caused the recent acute spinal cord compression based on neurological examination and magnetic resonance imaging (MRI) findings and to treat it with surgical decompression while ignoring the previously extruded or protruded discs [2,10,11]. Ventral slot decompression (VSD) is the standard surgical technique for cervical IVDE and IVDP. It provides direct access to the extruded or protruded disc material and is associated with good functional recovery [3,10,12]. Surgical techniques addressing multiple discs for spinal cord compression associated with IVDD at multiple sites have also been reported, such as VSD of multiple discs and dorsal laminectomy over several intervertebral spaces [10,11,13].

The prevalence of multiple-site IVDD leading to spinal cord compression in the cervical region varies considerably between studies. In a study evaluating adverse events associated with VSD, 1.5% of dogs had two sites of IVDE [3]. Another study analyzing the outcomes in non-ambulatory dogs with cervical IVDD and associated spinal cord compression reported that 3.1% of dogs had a disc herniation at two sites [14]. Studies from Asian countries have reported a higher prevalence. In a study by Hakozaki et al. evaluating the characteristics of cervical myelopathy associated with IVDD in small-breed dogs in Japan, approximately 26% had two or three affected discs [15]. Guo et al. reported an even higher prevalence of 34% in a study comparing outcomes between non-ambulatory dogs treated with single- or multiple-site VSD in Hong Kong [10]. The latter three studies used the term intervertebral disc herniation, and the type of IVDD was not further specified.

This study aimed to describe the outcomes and identify prognostic factors in dogs with an acute clinical presentation who were diagnosed with multiple sites of spinal cord compression from cervical IVDD via MRI and underwent VSD for the single acutely affected disc. In addition, these data were compared to dogs with a single IVDE who underwent VSD. We hypothesized that compared to dogs with a single IVDE, dogs with multiple-site cervical IVDD who underwent VSD for the single acute disc would have inferior outcomes.

## 2. Materials and Methods

### 2.1. Case Selection

Medical records of dogs presented at the National Taiwan University Veterinary Hospital between August 2008 and March 2023 were reviewed. Inclusion criteria were (a) dogs with ongoing cervical hyperesthesia and/or neurological signs indicating a C1-T2 myelopathy; (b) MRI evidence of single or multiple extradural compressive lesions consistent with cervical IVDE or IVDP; (c) a most recent IVDE or IVDP could be identified based on MRI in dogs with multiple sites of spinal cord compression associated with IVDD; (d) undergoing VSD over a single disc space; (e) surgical confirmation or high suspicion of IVDE by identifying the degenerative disc materials in the vertebral canal; and (f) follow-ups in our institute till achieving the successful outcome or at least 60 days postoperatively. In addition, the history should match one of the following patterns: (a) acute or sub-acute onset of clinical signs in a dog with no pre-existing signs of cervical myelopathy based on the owner’s observation; (b) had previous history potentially indicating cervical myelopathy and recovered with medical management, but acute or sub-acute clinical signs recurred at least one month later [9]; or (c) had chronic cervical hyperesthesia and/or mild neurological signs and recently experienced a drastic deterioration. Exclusion criteria were (a) a most recent IVDE or IVDP could not be identified on MRI in dogs with IVDE or IVDP at multiple sites; (b) VSD at multiple discs; (c) concurrent diseases interfering with the evaluation of neurological function; (d) incomplete medical records; (e) a diagnosis of hydrated nucleus pulposus extrusion; (f) reason for euthanasia or cause of death unrelated to the original cervical myelopathy; or (g) lost to follow-up. All dog owners provided informed consent for the use of the clinical data in this study.

### 2.2. MRI Investigation and Surgical Procedure

MRI of the cervical spine was performed under general anesthesia using a 0.2 T unit (Vet-MR, Esaote, Genova, Italy) or a 1.5 T unit (Ingenia-S, Philips Healthcare, Best, The Netherlands; Signa Creator, General Electric Healthcare, Waukesha, WI, USA). T2- and T1-weighted images in the sagittal and transverse planes were obtained for all dogs. To help distinguish acute from chronic spinal cord compression associated with IVDD, T2-myelo or post-contrast T1-weighted images were obtained in some dogs [1,16,17]. IVDD causing spinal cord compression was diagnosed based on the following criteria: extradural spinal cord compression at the intervertebral disc level, disc degeneration, and narrowing of the disc space. Each affected disc was further classified as IVDE or IVDP [18,19,20]. If multiple discs were affected, the most recent disc causing acute spinal cord compression was identified by comparing the degree of spinal cord swelling among all affected discs on transverse and sagittal T2-weighted and/or sagittal T2-myelo images when available [1,21]. Additional factors were evaluated as supporting information for the acute disc site, including IVDE, contrast enhancement of the extradural compressive material, and clinical evidence of lateralization that agreed with the MRI findings [1,16,17,22,23]. Concerning its association with IVDP, intraparenchymal hyperintensity adjacent to the affected disc on T2-weighted images was not used as supporting information for the acute disc [18]. Standard VSD was performed at the identified site by a board-certified neurologist or neurology resident [24]. Details of postoperative care varied between dogs based on individual patient conditions and the clinician’s decision but typically involved opioid analgesics and/or non-steroidal anti-inflammatory drugs for pain management, perioperative antibiotics, intravenous fluid therapy, balanced enteral nutrition by oral intake, and cage rest. Postoperative antibiotics were given when the patient had a concurrent infection, such as a urinary tract infection, and guided by culture and susceptibility testing when possible. Basic rehabilitation protocols included massage, passive range of motion, weight-bearing, and assisted walking when appropriate. Other rehabilitation techniques varied among patients.

### 2.3. Data Collection

The following clinical data were obtained from the medical records of each dog: breed, age, sex, pattern of history, preoperative neurological status, VSD site, and postoperative neurological status. Dog breeds were categorized as chondrodystrophic or non-chondrodystrophic [15,25]. The duration from the onset of clinical signs to the time of surgical decompression (D1) was recorded. In addition, the duration from the onset of non-ambulatory status to the time of surgery (D2) was documented. If the dog remained ambulatory preoperatively, the duration from deterioration to the severity at which the surgery was performed to the time of VSD was obtained. The neurological examination was performed by a board-certified neurologist or neurology resident in our institute on the day of the surgery (preoperative neurological status), daily during postoperative hospitalization, and on re-visits at the neurology service. For data analysis, the neurological status was recorded using the following grading system: grade 1, cervical pain; grade 2, ambulatory tetraparesis and/or proprioceptive ataxia; grade 3, non-ambulatory tetraparesis; grade 4, tetraplegia; and grade 5, tetraplegia with neurological hypoventilation [9,15,26,27]. For grades 3–5, if the neurological deficits varied between the limbs, the lower grade was chosen to represent the neurological status.

Two time points were set to determine the outcome status: the postoperative 30-day and the last re-examination at the neurology service (the overall outcome). A successful outcome was defined as recovery of independent ambulation and absent cervical hyperesthesia for a preoperatively non-ambulatory dog, gait improvement and absent cervical hyperesthesia for a preoperatively ataxic or ambulatory paretic dog, or the resolution of the cervical hyperesthesia for a dog presenting this sign as the only chief complaint preoperatively. Recovery time was defined as the duration from surgical decompression to when a successful outcome was first observed on neurological examination.

### 2.4. Spina Cord Compression Measurement

**Figure 1:**
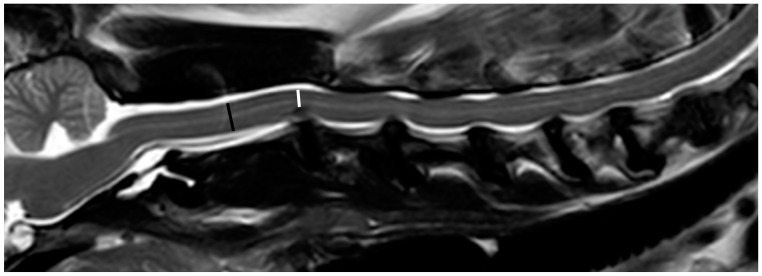
Midline sagittal T2-weighted image obtained with a 1.5 T MRI demonstrating measurement of spinal cord compression at the C2/C3 disc. The black line represents the spinal cord height at the middle level of the C2 vertebra, and the white line represents the spinal cord height under maximal compression at the C2/C3 disc. The spinal cord compression at C2/C3 disc is measured as 35%, classified as moderate severity.

A single examiner (YPC) reviewed all images and performed all measurements (RadiAnt DICOM Viewer 2021.2, Medixant, Poznan, Poland). The severity of spinal cord compression at each extruded or protruded disc was determined. All measurements were repeated three times to obtain the mean values. Owing to the imaging quality of the 0.2 T MRI, accurately measuring the spinal cord area in transverse T2-weighted images was considered difficult. Therefore, spinal cord compression was determined by measuring the spinal cord height (SCH) in the midline sagittal T2-weighted image by the following formula [16,28], if there was mainly ventral spinal compression (Figure 1):

$$Spinal\;cord\;compression=\left(1-\frac{SCH\;under\;maximal\;compression}{SCH\;at\;the\;middle\;level\;of\;the\;cranial\;vertebra}\right)\times 100\%$$

For lateralized disc herniation, the measurement was conducted using the transverse T2-weighted images only if the transverse images were perpendicular to the long axis of the spinal cord and the transverse image at the mid-level of the cranial vertebra was available. Otherwise, the dogs were excluded from the study. For each IVDE or IVDP, the severity of spinal cord compression was further categorized using a grading system: mild, <25%; moderate, 25 to <50%; and severe, ≥50%.

### 2.5. Statistical Analysis

The descriptive statistics for continuous variables are presented as mean and SD for normally distributed variables and as the median and interquartile range (IQR) for variables with a non-normal distribution. The frequencies of occurrence were reported for categorical and ordinal variables.

For dogs with multiple sites of spinal cord compression from IVDD, statistical analysis was performed to identify factors associated with the 30-day and overall outcomes, including age, sex, duration of clinical signs (D1 and D2), preoperative neurological status, the number of affected discs, the presence and the number of affected discs causing severe spinal compression, the spinal compression severity at the disc site that underwent VSD, and the presence of other affected discs causing similar (difference in spinal compression <5%) or more severe spinal compression than the site underwent VSD. Continuous variables were analyzed using the t-test or Mann–Whitney *U* test, depending on the Shapiro–Wilk test results. Categorical and ordinal variables were analyzed using the Chi-square or Fisher’s exact test.

Variables were also compared between dogs with multiple-site IVDD (IVDE or IVDP) and dogs with a single IVDE, including age, sex, the duration of clinical signs (D1 and D2), preoperative neurological status, the spinal compression severity at the disc site receiving VSD, the 30-day recovery outcome, the overall recovery outcome, and the recovery days. Continuous variables were analyzed using the t-test or Mann–Whitney *U* test, depending on the Shapiro–Wilk test results. Categorical and ordinal variables were analyzed using the chi-squared or Fisher’s exact tests. The association between the total number of affected discs and recovery outcomes was further analyzed. The dogs were categorized into three groups based on the total number of affected discs: one, two, and ≥3 discs. The 30-day and overall outcomes were compared among the three groups using the Chi-squared or Fisher’s exact tests. The recovery time was analyzed using a one-way ANOVA or the Kruskal-Wallis test, depending on the Shapiro–Wilk test results.

All statistical analyses were performed using IBM SPSS Statistics for Windows (version 25.0; IBM Corp., Armonk, NY, USA). Statistical significance was set at *p* < 0.05.

## 3. Results

Based on the MRI findings, 83 dogs diagnosed with cervical IVDE or IVDP underwent VSD during the study period. Twenty dogs were excluded for various reasons, including that VSD was performed at multiple discs in one surgery due to the inability to identify the most recent IVDD (n = 6), concurrent disease interfering with neurological evaluation (n = 4), incomplete medical records (n = 3), MRI findings suggestive of hydrated nucleus pulposus extrusion (n = 2), euthanasia due to other causes (n = 3), and being lost to follow-up (n = 2). Of the remaining 63 dogs, 40 were classified as having IVDE or IVDP at multiple sites, and 23 were classified as having a single IVDE.

### 3.1. Clinical Presentation, MRI Findings, and Outcomes in Dogs with Multiple-Site IVDD and Underwent VSD for the Single Acute Disc

The descriptive data of 40 dogs with cervical IVDE or IVDP at multiple sites are summarized in Table 1. The mean age was 10.5 ± 2.4 years, ranging from five to 16 years. Male dogs were more frequently presented. Maltese was the most common breed (20%) among the 14 breeds presented. Twenty dogs were chondrodystrophic, and 20 were non-chondrodystrophic. Regarding the pattern of history, most dogs had no pre-existing signs indicating cervical myelopathy prior to the acute or sub-acute clinical signs (n = 32, 80%). Seven dogs (17.5%) had a previous history potentially indicating cervical myelopathy but recovered with medical management 3–36 months ago before the recent acute neurological signs. One dog had at least a 6-month history of mild weakness prior to the acute and marked ambulatory tetraparesis. For the duration of clinical signs, the medians of D1 and D2 were 19 and 13.5 days, respectively. Non-ambulatory tetraparesis was the most common presentation prior to surgery (57.5%). In dogs with the preoperative status of non-ambulatory tetraparesis or tetraplegia, the medians of D1 and D2 were 13 (IQR 3–29.75) and 4 (IQR 2.25–14) days, respectively.

**Figure 2:**
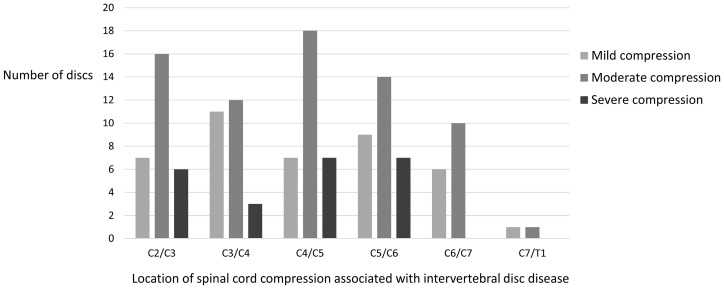
The distribution of affected discs and concomitant spinal cord compression severity in dogs with multiple-site cervical intervertebral disc disease.

IVDE or IVDP was identified in 135 discs among the 40 dogs. The number of affected discs in each dog ranged from 2 to 6, with a median of 3 (IQR 2–4). In 26 dogs, the acute IVDE was accompanied by various numbers of chronic IVDP. In seven dogs, in addition to the acute IVDE and various numbers of chronic IVDP, one sub-clinical IVDE was observed. The acute disc was classified as IVDP on MRI in another seven dogs. Features for identifying the acute disc site in individual patients are summarized in Table 2. The location of spinal cord compression associated with IVDD is illustrated in Figure 2. Moderate spinal compression (25–50% compression) was observed in 53% of all affected discs (71/135) and 85% of all dogs (34/40). Severe spinal compression (≥50% compression) was observed in 17% of all affected discs (23/135) and 50% of all dogs (20/40). Furthermore, 17 dogs had one affected disc, and three dogs had two affected discs, leading to severe spinal compression.

Approximately two-thirds of the dogs (26/40) with multiple sites of IVDE or IVDP underwent VSD at either the C4/C5 or C5/6 intervertebral disc spaces. In 12 dogs, other affected discs caused similar (n = 8) or more severe (n = 4) compression than that at the surgical site.

The median recovery time in dogs with multiple sites of IVDE or IVDP was seven days. The 30-day and overall outcomes were documented as successful in 80% and 97.5% of dogs, respectively. One dog was euthanized two days postoperatively due to neurological deterioration.

### 3.2. Prognostic Factors Associated with Outcome in Dogs with Multiple-Site IVDD and Underwent VSD for the Single Acute Disc

Several factors were evaluated for their association with the 30-day outcome, including age, sex, the duration of clinical signs, preoperative neurological status, the number of affected discs, the presence and number of affected discs causing severe spinal compression, the spinal compression severity of the disc underwent VSD, and having other affected discs causing similar or severer compression than that at the surgical site. However, none of the factors was associated with the 30-day outcome. An analysis was not conducted for the overall outcome due to the low number of dogs with an unsuccessful overall outcome.

### 3.3. Clinical Presentation, MRI Findings, and Outcomes in Dogs with Single IVDE and Underwent VSD

The mean age for the 23 dogs with a single IVDE was 8.6 ± 3.1 years (2.5 to 14 years). Most dogs were male (82.6%). Maltese, beagle, and miniature dachshund were the most common breeds (four dogs of each breed). Ten dogs were chondrodystrophic, and 13 were non-chondrodystrophic. The median duration of clinical signs D1 and D2 was 14 and 6 days, respectively. Nonambulatory tetraparesis was the most common neurological severity prior to surgery (47.8%). In dogs with the preoperative status of non-ambulatory tetraparesis or tetraplegia, the medians of D1 and D2 were 12 (IQR 4.5–48.5) and 4.5 (IQR 2–15.75) days, respectively.

Common sites for a single IVDE were C2/C3, C4/C5, and C5/C6. The spinal cord compression was often moderate or severe (47.8% and 43.5% of dogs, respectively). The 30-day and overall recovery rates were 91.3% and 100 %, respectively. The median recovery time was seven days. Descriptive data for dogs with a single IVDE are summarized in Table 1.

### 3.4. Comparison between Dogs with Multiple-Site IVDD and Dogs with Single IVDE

The mean age of the dogs in the multiple-site IVDD group was significantly higher than that of those in the single-IVDE group (Table 1). For all other variables, no significant difference was detected between the two groups, including sex, duration of clinical signs, preoperative neurological status, the spinal compression severity of the disc that underwent VSD, the 30-day outcome, the overall outcome, and recovery days. Further analysis revealed no association between the total number of affected discs and 30-day outcomes, overall outcomes, or recovery days (Table 3).

## 4. Discussion

In this study, we described the outcomes in dogs with cervical IVDE or IVDP at multiple sites who underwent a single VSD for the acute disc site and searched for prognostic factors in this population. Following surgery, 80% of the dogs recovered within 30 days. Overall, 97.5% of the dogs recovered successfully. The median recovery time was seven days. There is limited research on recovery outcomes following this management strategy. Hakozaki et al. described cervical intervertebral disc herniation in 187 small-breed dogs that underwent surgery, of which 253 discs were affected [15]. Of the 137 dogs with recovery times available for analysis, 36 had two or three affected discs. The authors did not describe the details of the surgical decision but reported that the five dogs in their study required a second surgical procedure because of cervical disc herniation involving other sites. Therefore, it may be presumed that most dogs with multiple affected discs in their study initially underwent decompressive surgery for a single disc. Other studies and case reports describing multiple sites of spinal cord compression associated with cervical IVDD have mainly focused on using surgical management to address multiple discs [10,13,14].

In dogs with acute onset of cervical myelopathy, we used the degree of spinal cord swelling on MRI to identify the acute disc site among all affected discs with concomitant spinal cord compression. Features supporting the identification of the acute disc included IVDE, contrast enhancement of the extradural compressive material, and clinical evidence of lateralization that agreed with the MRI findings. Although intraparenchymal T2-weighted hyperintensity was reported in up to 65% of non-ambulatory paraparetic or paraplegic dogs with thoracolumbar IVDE [21,29], this feature was also commonly observed in chronic IVDP [1]. Moreover, intraparenchymal signal intensity changes were significantly associated with IVDP in one study investigating MRI features to distinguish between IVDE and IVDP [18]. Therefore, we did not use this feature as supporting information for acute disc site. In the present study, the most common scenario was an acute IVDE with multiple previous IVDPs and/or one sub-clinical IVDE, which counted for 82.5% of dogs. However, in the remaining seven dogs, the acute disc was classified as IVDP along with other chronic IVDP on imaging. A 0.2 T MRI machine was used in these seven patients. Degenerative disc materials and fragments resembling layers of the annulus were retrieved from the vertebral canal during surgery in these dogs. Due to the small window and the nature of the ventral approach in VSD, determining whether the annulus was completely ruptured to distinguish between IVDE and IVDP was difficult [25]. However, we only included dogs with an acute presentation in this study, contrary to the typical clinical manifestation of slow-progressive myelopathy in IVDP. Studies investigating the MRI characteristics to distinguish between IVDE and IVDP were conducted in dogs with thoracolumbar discs utilizing high-field MRI [18,19,20]. It is uncertain about the accuracy of applying the same MRI characteristics to cervical discs using low-field MRI. We postulated that the imaging quality of low-field MRI might impact the accuracy of differentiating the disc type. Another speculation was that these dogs might experience an acute extrusion on a previously protruded disc. We, therefore, used spinal cord swelling as the primary criterion for identifying the acute disc and IVDE as supporting information. Further study is warranted to evaluate the accuracy of these criteria for identifying the acute disc site among multiple sites of IVDE or IVDP. Nevertheless, considering the surgical confirmation of degenerated disc materials in the vertebral canal and the fair clinical outcomes, the MRI criteria reported here seemed to be a viable approach.

In the present study, the number of affected discs, the presence and number of affected discs causing severe spinal compression, and the presence of other affected discs causing similar or more severe compression than that at the surgical site did not influence the 30-day outcome in dogs with multiple sites of IVDE or IVDP. After combining the data from dogs with a single IVDE, further analysis revealed no evidence that the total number of affected discs influenced the 30-day and overall outcomes or recovery time. In a study describing the characteristics of small-breed dogs with a cervical disc herniation that underwent surgery, Hakozaki et al. reported that the mean recovery time for dogs with three affected discs was significantly longer than that for dogs with only one affected disc [15]. Our findings contradict those of Hakozaki et al. However, differences in data collection made comparison between the two studies difficult. We determined spinal cord compression by comparing the SCH under maximal compression with the SCH at the middle level of the cranial vertebra in a midline sagittal T2-weighted image. In contrast, in Hakozaki’s study, diagnoses were made via myelography, CT, or MRI, and the affected discs were subjectively identified based on spinal cord compression. The sensitivity of detecting spinal cord compression associated with IVDD differed among the three modalities [20,30,31]. Considering the differences in imaging modalities and definitions of the affected disc, the results may not be comparable between the two studies.

Another difference between Hakozaki’s study and ours was the proportion of dogs with various numbers of affected discs and the proportion of non-chondrodystrophic breed dogs. In their study, the number of dogs with one affected disc was much greater than that in the other two groups (101, 27, and 9 dogs with one, two, and three affected discs, respectively) [15]. In contrast, the number of dogs was relatively similar among the three groups in our study (23, 11, and 29 dogs with one, two, and ≥3 affected discs, respectively). We combined all cases with ≥3 IVDE or IVDP into one group, which might have contributed to its greatest proportion. The low number of dogs with a single IVDE in our study may be attributed to the inclusion criteria for using MRI as the diagnostic modality. If IVDE was considered the top differential diagnosis and mineralized disc materials were detected in radiographs, the choice of MRI or CT for further investigation at our institute depends on the clinical judgment of the individual clinician. However, CT was often chosen in the aforementioned situation due to various considerations, such as the owner’s financial concerns, anesthesia time, and the imaging quality of the low-field MRI. Only dogs with MRI examinations were included in this study because non-contrast CT might have underestimated the presence of IVDP and IVDE when the extruded disc materials were not mineralized [1,20]. However, this would influence case collection. In our study, 50% of the dogs were non-chondrodystrophic, likely reflecting the aforementioned situation. In contrast, only 29% of the dogs in Hakozaki’s study were non-chondrodystrophic [15]. They also reported that non-chondrodystrophic dogs had a higher mean number of affected discs and a longer mean recovery time than chondrodystrophic dogs. We use the same reference to define the chondrodystrophic status as Hakozak’s study for results comparison. The different proportions of non-chondrodystrophic dogs might partially explain the different results regarding how the number of affected discs influenced the recovery outcome between the two studies.

Although surgical decompression is considered one of the standard management methods for cervical IVDE in dogs, inconsistent results have been reported regarding the association between the degree of preoperative spinal cord compression and recovery outcome [12,23,26]. Moreover, reports describing the influence of residual spinal cord compression on clinical outcomes are also contradictory [12,32]. Bottcher et al. evaluated the effect of VSD on spinal cord compression in dogs with single IVDD using CT-myelography. Although approximately 77% of dogs had residual compression, it did not affect their long-term neurological outcomes [12]. In contrast, Tirrito et al. reported that satisfactory decompression, assessed using MRI, was associated with better recovery outcomes [32]. These results reflect the complexity of spinal cord injury and functional recovery. In addition, spinal cord compression may be observed on MRI examinations of dogs without neurological signs, further complicating decision-making when managing dogs with compressive lesions [2,33,34]. Currently, the most appropriate management for dogs with an acute presentation but showing multiple sites of cervical IVDE or IVDP on MRI remains uncertain. In the present study, we describe the outcomes in this population following VSD for the single acute disc. The rationale for this management is partially supported by an in vivo experimental mouse study [35]. Using an extradural spacer to create chronic spinal cord compression and an impactor system to create an acute contusive injury, this study demonstrated that asymptomatic pre-existing cord compression did not impair neurological recovery after an acute spinal cord injury. Spinal cord blood flow decreased immediately following the compressive lesion but recovered completely in the chronic phase. Following acute contusive injury, there were no significant differences in the hemodynamics of spinal cord blood flow or neurological recovery between mice with pre-existing spinal cord compression and the control group. Several factors, such as the type of spinal cord injury and different responses to spinal cord injury between species, should be considered before translating this information to the clinical setting of canine IVDD and concomitant spinal cord compression. Nevertheless, this may explain why the outcomes in dogs with multiple sites of IVDE or IVDP were similar to those in dogs with a single IVDE in our study.

Ideally, we should compare the results with the outcomes in dogs with multiple sites of spinal cord compression from IVDD that undergo VSD at multiple discs. However, this investigation was not conducted due to the scarcity of cases managed using this approach at our institute. Guo et al. evaluated the outcomes of non-ambulatory dogs with multiple sites of cervical IVDD that underwent multiple-site VSD [10]. IVDE or IVDP was not differentiated, and they categorized all cases as disc herniation. Using a modified surgical technique to create a smaller slot, 62 dogs underwent multiple-site VSD, almost exclusively performed by a single neurologist. Due to the difference in inclusion criteria, the recovery rate could not be compared. However, they reported a median time to ambulation of 4 days, which was superior to our results. Interestingly, the functional recovery of dogs with multiple-site VSD was similar to that of dogs with a single disc herniation that underwent VSD in Guo’s study. This potentially indicates that the outcome in dogs following VSD in their study was excellent in general, instead of suggesting that multiple-site VSD is a better treatment option than a single-site VSD at the acute disc for dogs with multiple sites of IVDE or IVDP. Although no instability was reported in Guo’s study or another case report [10,11], little is known about the biomechanical characteristics of multiple or consecutive VSD. A recent study reported that microendoscopic dorsal laminectomy for multi-level cervical IVDP in dogs was also feasible and provided good long-term clinical outcomes [13]. Further research is warranted to determine the most appropriate management strategy for dogs with acute presentations but diagnosed with multiple sites of cervical IVDE or IVDP.

The duration of clinical signs in the present study appeared longer than in previous studies, even for dogs presenting non-ambulatory tetraparesis or tetraplegia prior to the surgery [14,23]. A similar pattern was observed in dogs with multiple sites of IVDE or IVDP and in dogs with a single IVDE. In our culture, many owners are reluctant to perform procedures involving general anesthesia and invasive procedures and tend to choose medical management. In addition, traditional Chinese veterinary medicine is a popular choice for many owners locally. Due to the diversity and incomplete information, treatment prior to the surgery was not analyzed. The duration of clinical signs, including D1 and D2, did not influence the 30-day outcome in dogs with multiple sites of IVDE or IVDP. However, its impact remains uncertain if the data contains a larger proportion of dogs undergoing VSD at an earlier stage.

In addition to the aforementioned bias in case collection and considering its retrospective nature, this study has other potential limitations worth noting. During the study period, two board-certified neurologists and two residents were involved in patient management and neurosurgery. Standardized neurological assessments and surgical training were performed. However, subtle variations may still exist among clinicians. Postoperative MRI is not routinely performed at our institute. The effectiveness of spinal cord decompression could not be objectively evaluated. Kinematic MRI was not performed; therefore, the influence of dynamic compressions remained unclear in dogs with multiple sites of IVDE or IVDP. Recovery status was determined based on the neurological examination of the medical records. A gap between the actual and recorded recovery days likely existed, which might be more prominent in patients with a slower recovery. Either a low- or high-field MRI was used in the present study. Different imaging qualities create a bias. Concerning the accuracy of measuring the spinal cord area in transverse images of low-field MRI, we measured spinal cord compression via spinal cord height. This design may have partially reduced the bias due to the different imaging qualities of the two MRI machines. The small sample size likely limited the power of the study to detect statistically significant associations. A prospective study with a larger sample size, involving fewer clinicians, using high-field MRI for diagnosis, and applying a more objective scoring system to assess spinal cord function (e.g., stepping and coordination scores) at designated time points would be ideal for overcoming the bias mentioned above.

## 5. Conclusions

In dogs with an acute presentation but diagnosed with multiple sites of IVDE or IVDP, the 30-day and overall recovery rates were 80 and 97.5%, respectively, following VSD at the single acute disc. No difference was detected in recovery time or outcomes between dogs with multiple sites of IVDE or IVDP and dogs with a single IVDE. There was also no evidence suggesting that the total number of affected discs influences recovery outcomes. Based on our results, if the acute disc could be identified on MRI, ventral slot decompression for a single acute disc site is a viable management approach for dogs with an acute presentation but diagnosed with multiple sites of spinal cord compression associated with IVDD.

## Figures and Tables

**Table 1:** Descriptive data of dogs with cervical IVDE or IVDP at multiple sites and dogs with single cervical IVDE.

Variables	Multiple IVDE/IVDP n = 40	Single IVDE n = 23	*p*-Value *
Breed, n (%)			
Maltese	8 (20)	4 (17.4)	
Beagle	5 (12.5)	4 (17.4)	
Miniature dachshund	5 (12.5)	4 (17.4)	
Shih-tzu	5 (12.5)	0 (0)	
Pekingese	3 (7.5)	0 (0)	
Chihuahua	3 (7.5)	2 (8.7)	
Whippet	2 (5)	0 (0)	
Pug	2 (5)	0 (0)	
Miniature schnauzer	2 (5)	0 (0)	
Yorkshire terrier	1 (2.5)	1 (4.3)	
Miniature pincher	1 (2.5)	0 (0)	
Miniature poodle	1 (2.5)	1 (4.3)	
Japanese Spitz	1 (2.5)	0 (0)	
Mixed	1 (2.5)	3 (13)	
Labrador retriever	0 (0)	2 (8.7)	
French bulldog	0 (0)	1 (4.3)	
Cavalier King Charles spaniel	0 (0)	1 (4.3)	
Sex, n (%)			0.37
Male	28 (70)	19 (82.6)	
Female	12 (30)	4 (17.4)	
Age, y, mean (SD)	10.5 (2.4)	8.6 (3.1)	<0.01
D1, median (IQR)	19 (5.25–30.75)	14 (6–38)	0.47
D2, median (IQR)	13.5 (3–20.75)	6 (3–16)	0.32
Neurological severity, n (%)			0.60
Grade 1 and 2	12 (30)	7 (30.4)	
Grade 3	23 (57.5)	11 (47.8)	
Grade 4	5 (12.5)	5 (21.7)	
Total number of affected discs, n (%)			
1	NA	23 (100)	
2	11 (27.5)	NA	
3	11 (27.5)	NA	
4	11 (27.5)	NA	
5	6 (15)	NA	
6	1 (2.5)	NA	
MRI, n (%)			0.46
0.2 T	28 (70)	14 (60.9)	
1.5 T	12 (30)	9 (39.1)	
Successful outcome, n (%)			
30-day outcome	32 (80)	21 (91.3)	0.30
Overall	39 (97.5)	23 (100)	0.29
Recovery days, median (IQR)	7 (3–18)	7 (4–13)	0.71
Follow-up days, median (IQR)	28 (12–62)	27 (15–57)	0.50

D1, duration from the onset of clinical signs to surgery; D2, duration from the onset of non-ambulatory status to surgery; IQR, interquartile range; IVDE, intervertebral disc extrusion; IVDP, intervertebral disc protrusion; NA, not applicable; SD, standard deviation. * Comparison between the two groups.

**Table 2:** In addition to extradural spinal cord compression associated with a degenerative disc on MRI, features for identifying the acute disc site in the 40 dogs with multiple-site intervertebral disc disease.

Features	Dog Number (%)
Spinal cord swelling, IVDE, contrast enhancement ^†^	19 (47.5)
Spinal cord swelling, IVDE	11 (27.5)
Spinal cord swelling	5 (12.5)
Spinal cord swelling, IVDE, contrast enhancement ^†^, lateralization *	2 (5)
Spinal cord swelling, contrast enhancement ^†^	2 (5)
Spinal cord swelling, IVDE, lateralization *	1 (2.5)

IVDE, intervertebral disc extrusion; ^†^ contrast enhancement of the extradural compressive material; * clinical evidence of lateralization agreed with the MRI findings.

**Table 3:** The recovery outcomes in dogs with various numbers of IVDE or IVDP.

	Number of Affected Discs			
	One Disc n = 23	Two Discs n = 11	≥3 Discs n = 29	*p*-Value
30-day successful outcome, n (%)	21 (91%)	10 (91%)	22 (76%)	0.35
Overall successful outcome, n (%)	23 (100%)	11 (100%)	28 (97%)	>0.9
Recovery day, median (IQR)	7 (4–13)	5 (2–10)	9.5 (3–19.5)	0.34

IQR, interquartile range; IVDE, intervertebral disc extrusion; IVDP, intervertebral disc protrusion.

## Data Availability

The data presented in this study are available from the corresponding author upon request.
